# Supplementary material for: Mycobacterium tuberculosis overcomes phosphate starvation by extensively remodelling its lipidome with phosphorus-free lipids
Source: Nat Commun. 2025 Nov 20;16:11317. doi: 10.1038/s41467-025-66437-w (PMC12722247; doi:10.1038/s41467-025-66437-w)
Supplement: Supplementary file 1 — Supplementary Information [file 41467_2025_66437_MOESM1_ESM.pdf]

Supplementary Figure 1

a

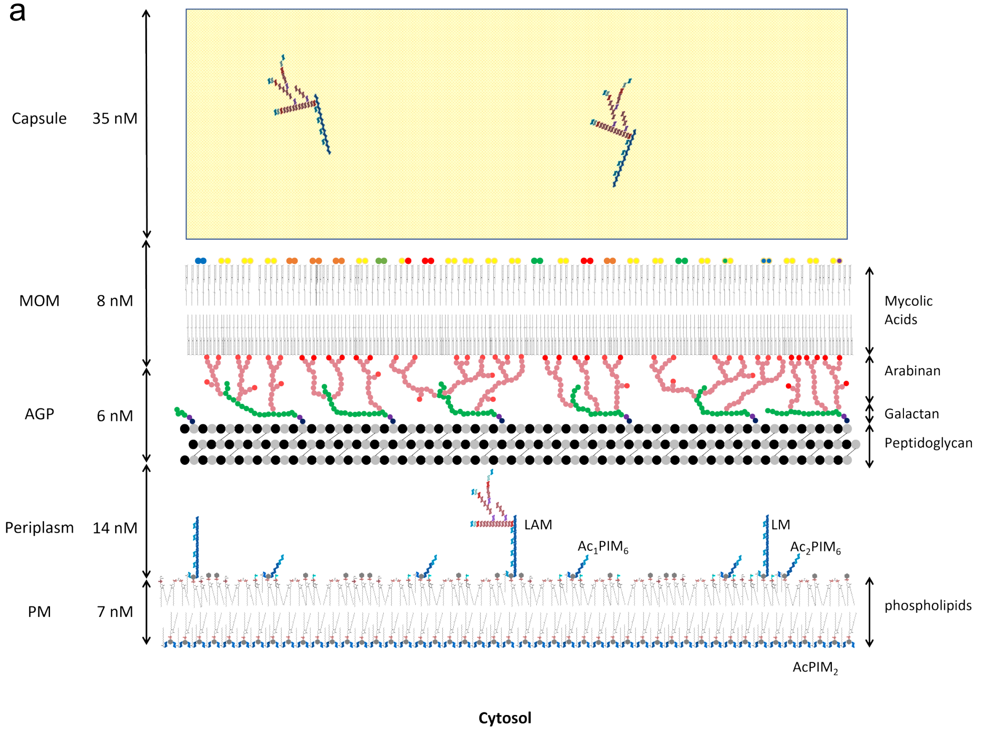

b

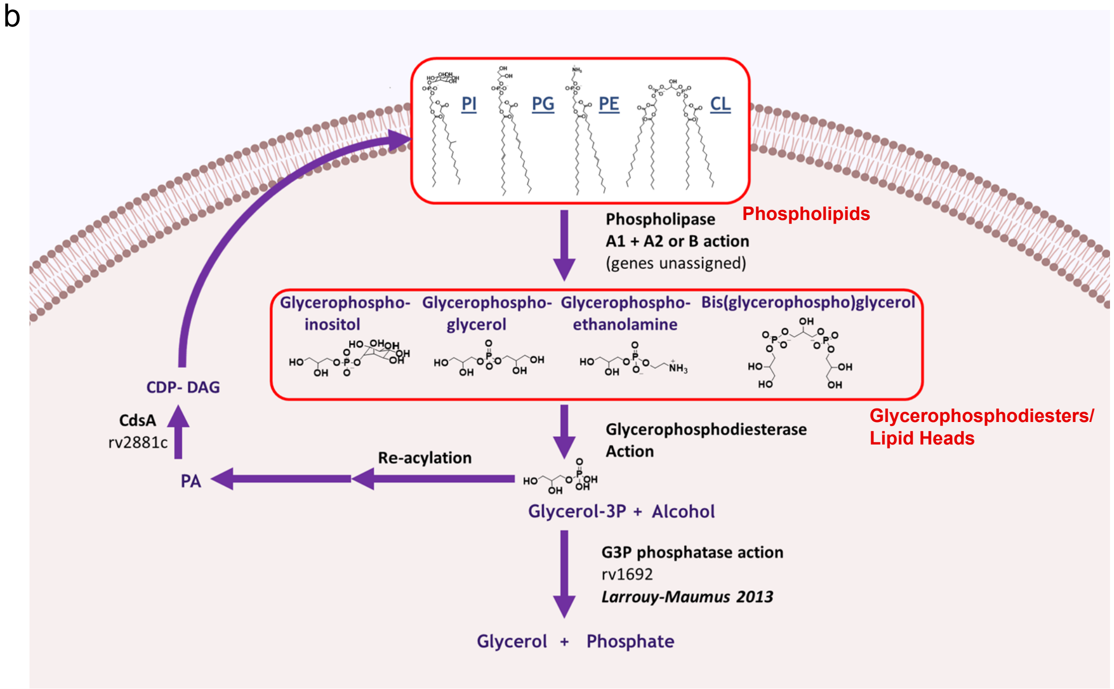

c

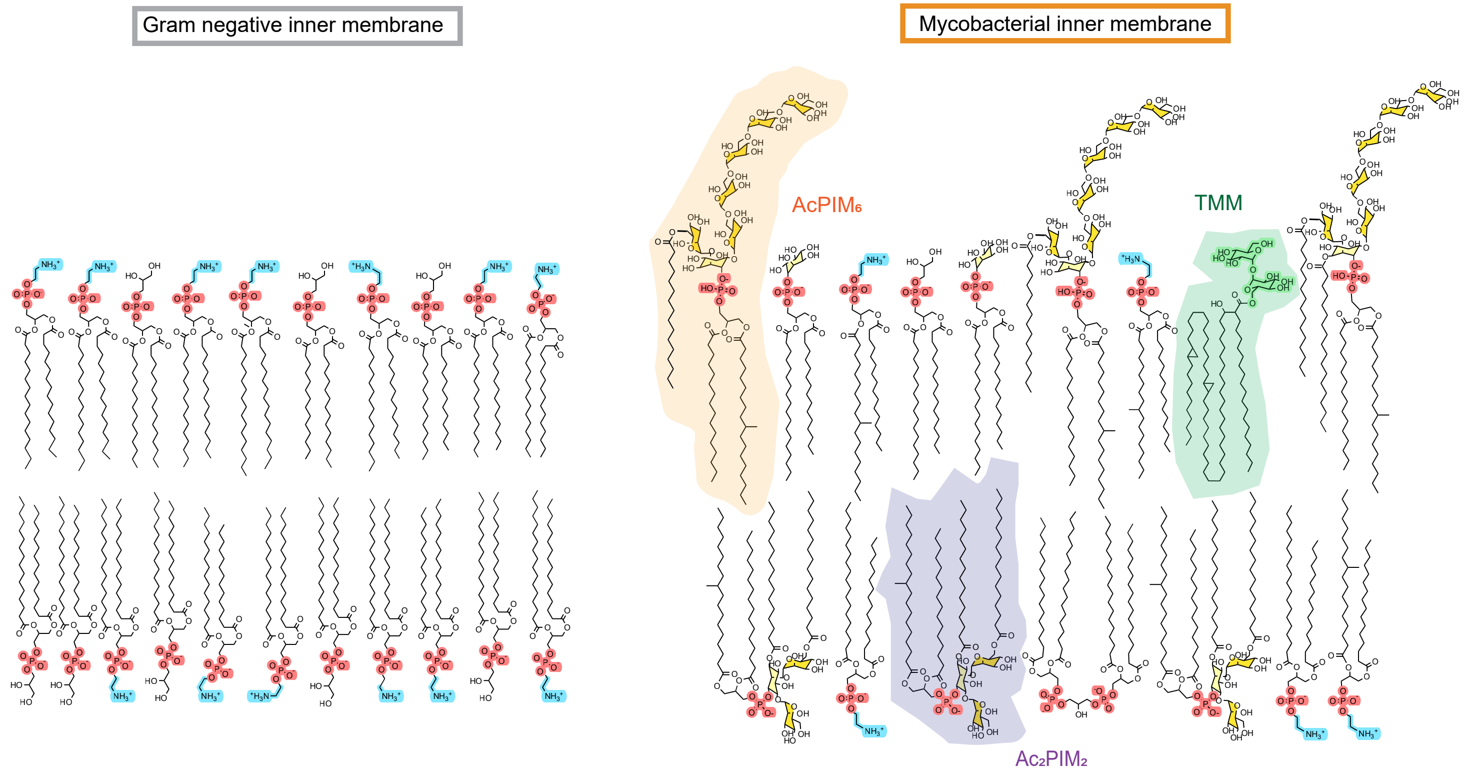

Supplementary Figure 2

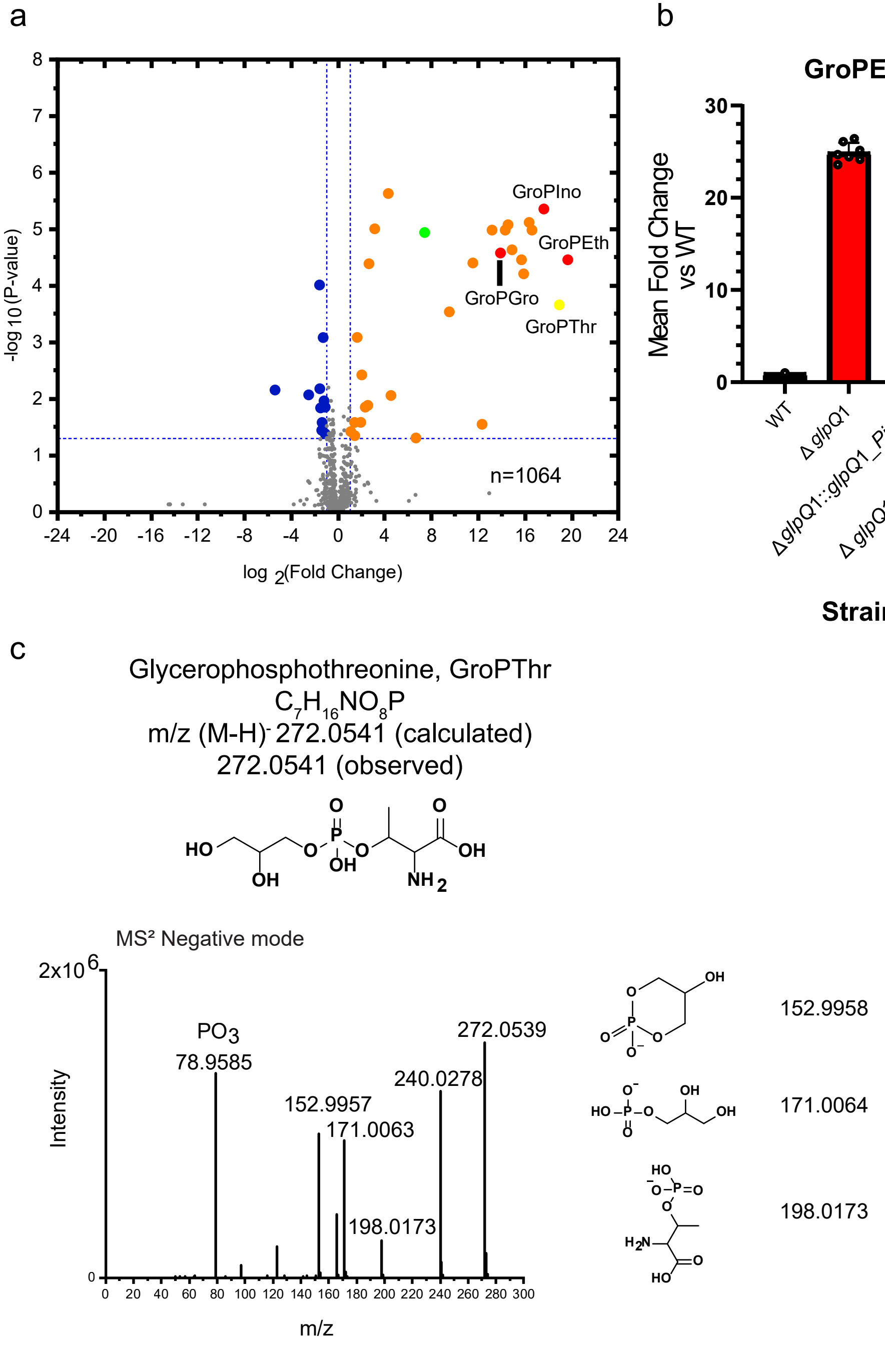

Supplementary Figure 3

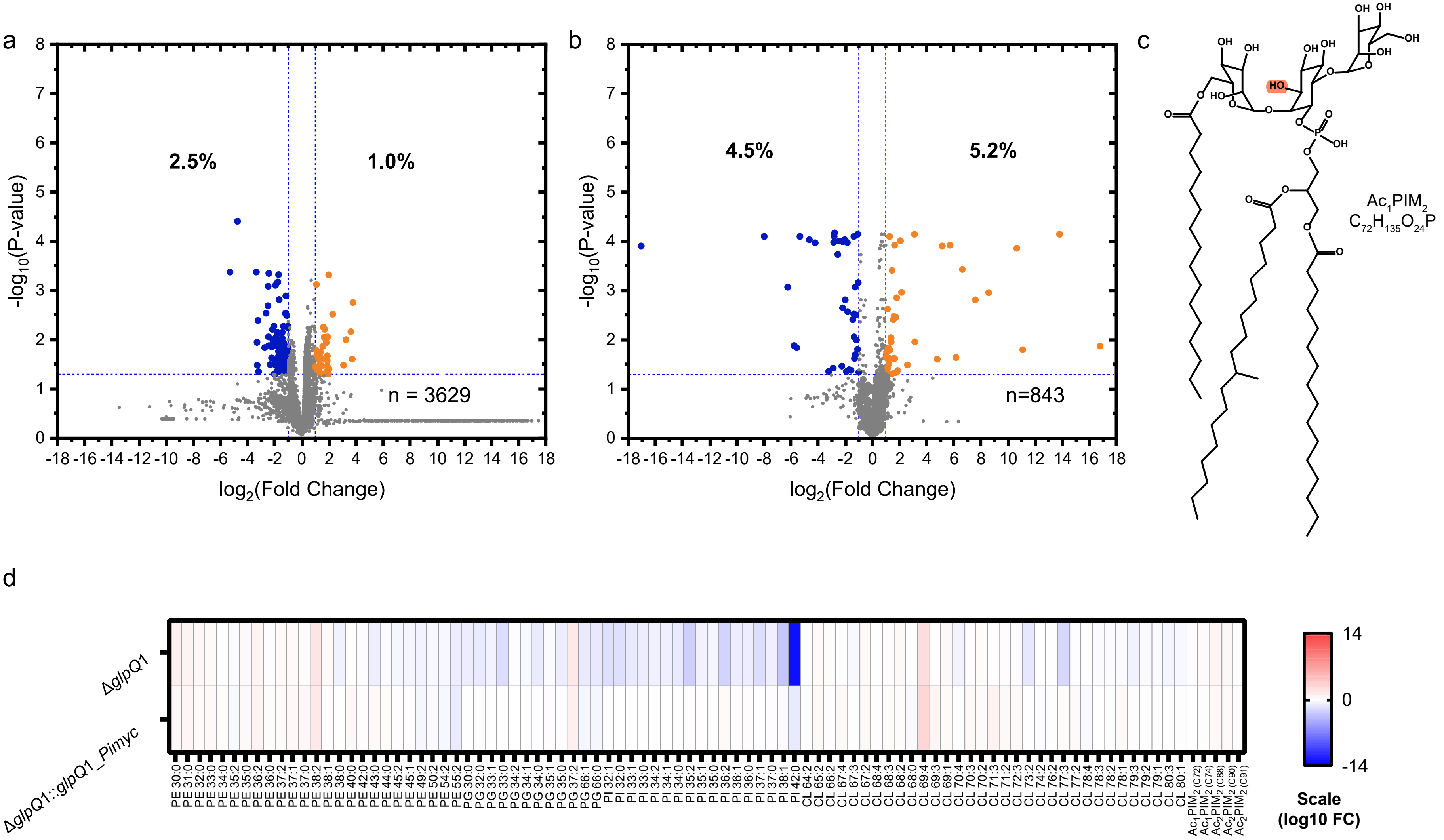

Supplementary Figure 4

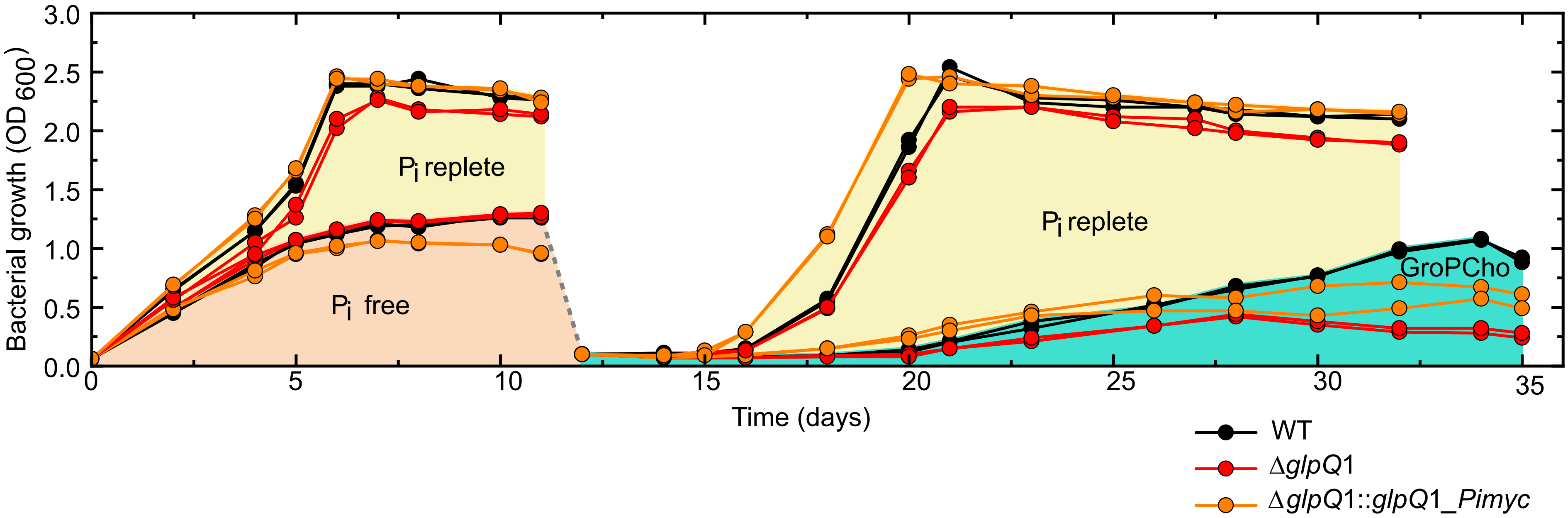

Supplementary Figure 5

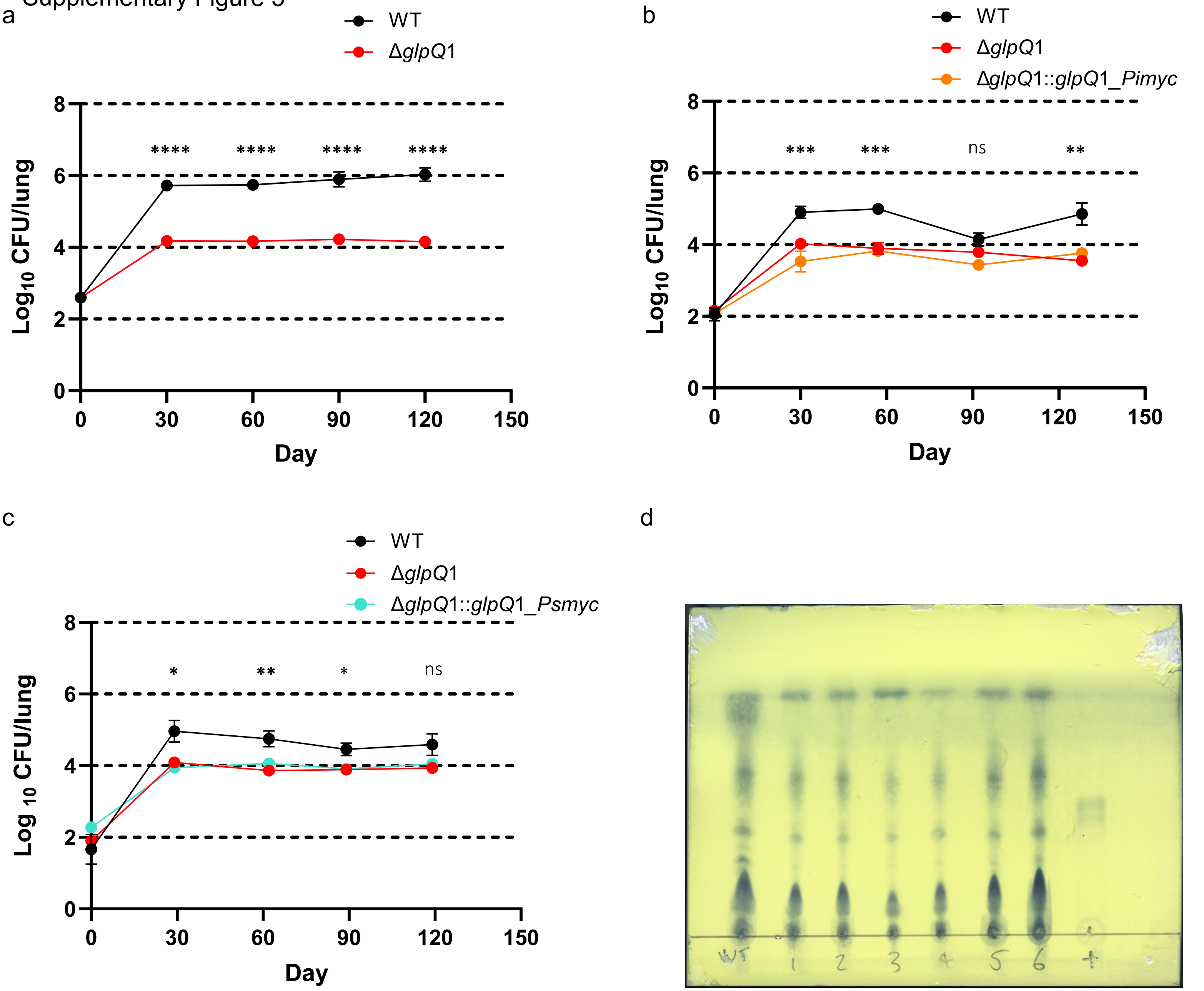

Supplementary Figure 6

a

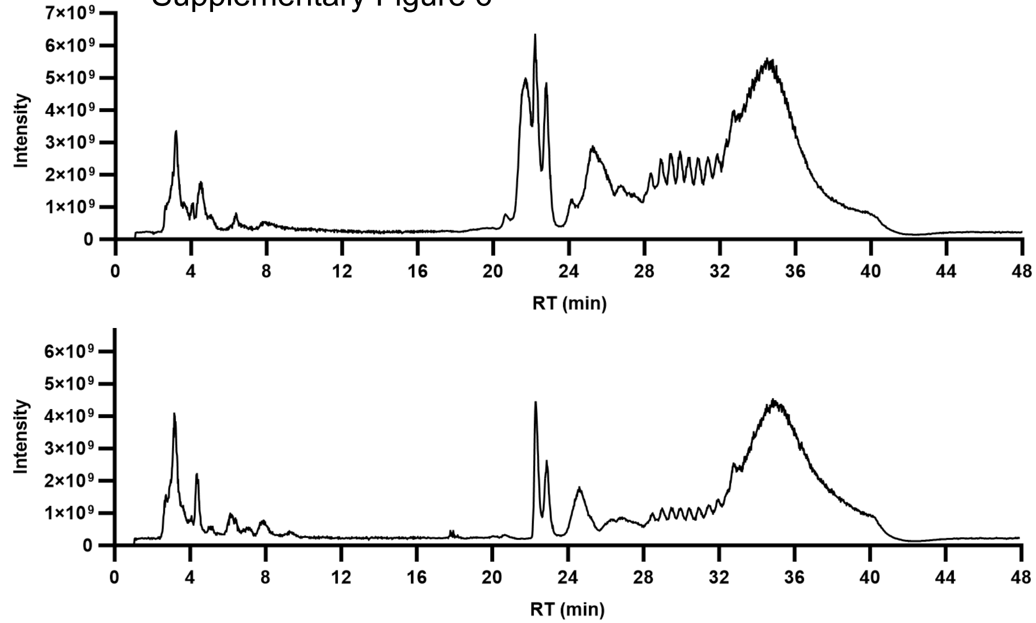

b

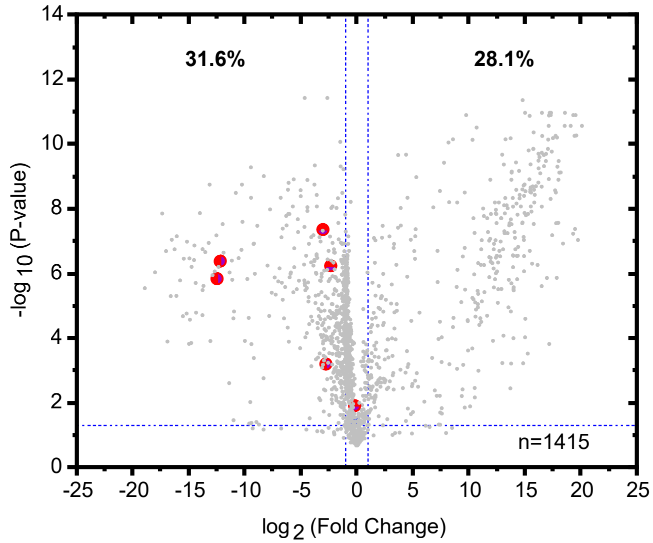

c

		Total area under curve (average across replicates)		Fold change	T-test
Class	no. of lipids	Pi free	Pi replete	Pi free / Pi replete	p value
PEs	17	9.49×10^{8}	3.88×10^{10}	0.024	7.2×10^{-11}
PGs	16	9.12×10^{8}	4.28×10^{8}	2.134	3.1×10^{-7}
PIs	8	1.92×10^{9}	8.84×10^{9}	0.217	9.4×10^{-4}
CLs	20	1.46×10^{10}	1.95×10^{10}	0.750	5.7×10^{-5}
All conventional PLs	61	1.84×10^{10}	6.75×10^{10}	0.272	1.1×10^{-10}
PIM	5	3.17×10^{9}	4.75×10^{9}	0.669	1.0×10^{-4}
MPM	4	2.37×10^{6}	4.23×10^{7}	0.056	4.8×10^{-8}
All PLs	70	2.16×10^{10}	7.23×10^{10}	0.298	1.1×10^{-10}

d

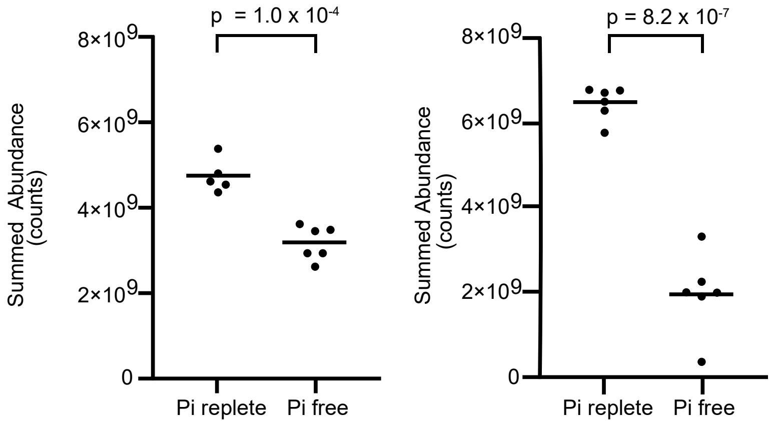

e

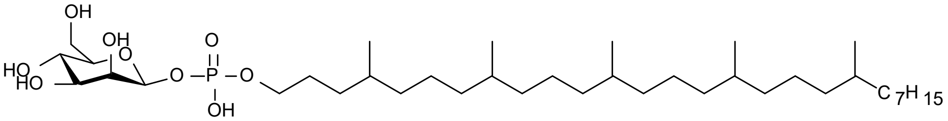

f

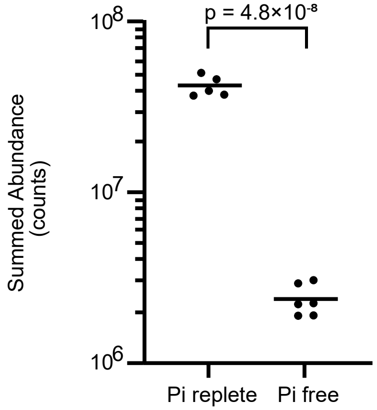

g

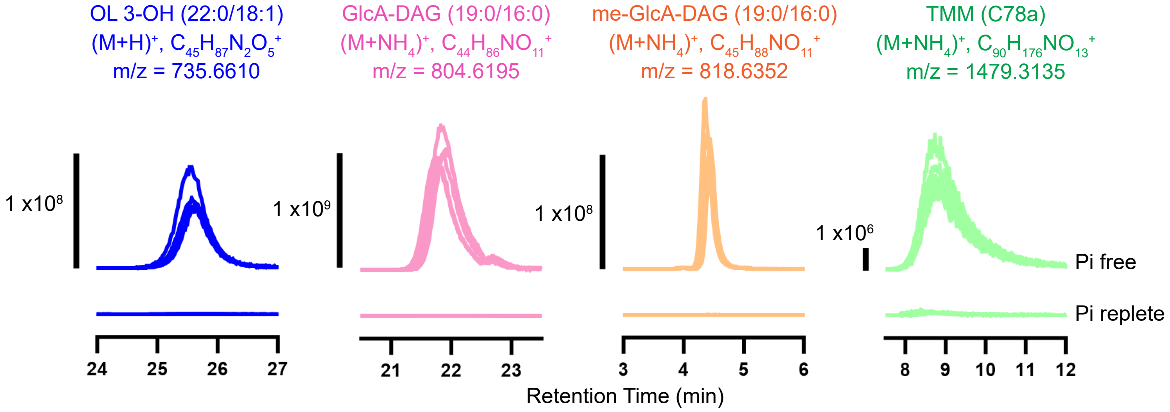

Supplementary Figure 7

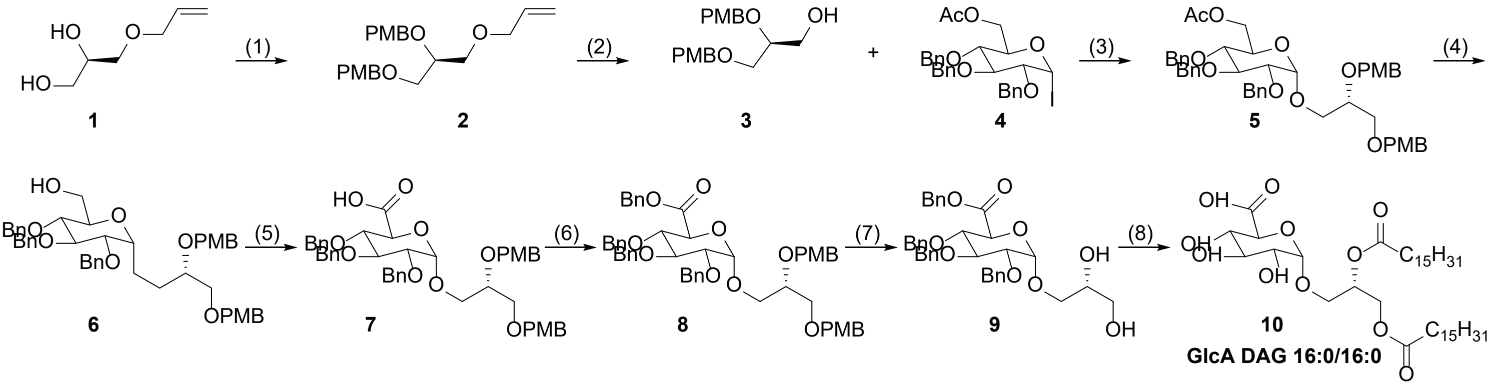

Reagents and conditions: (1) NaH 95%, DMF, p-methoxybenzyl chloride, TBAI, 71%; 2) $PdCl_2$, CuCl, DMF, 44%; (3) TBAI, 2,4,6-tri-tert-butylpyrimidine, CH_2Cl_2, 60%; (4) NaOMe in MeOH, CH_2Cl_2, 92%; (5) TEMPO, BAIB, CH_2Cl_2/H_2O, 88%; (6) benzyl alcohol, HBTU, DIPEA, DMAP, CH_2Cl_2, 34%; (7) CAN, MeCN/H_2O, 44%; (8) a. palmitic acid, COMU, DMAP, DMF; b. $Pd(OH)_2/C$, H_2, MeOH/THF, 8%.

SUPPLEMENTARY FIGURE LEGENDS

Supplementary Figure 1. Envelope lipids of Mtb. a: Model of the envelope of Mtb. The envelope comprises of two lipid bilayers, the plasma membrane (PM) of phospholipids and acylated phosphatidylinositol mannosides (AcPIMs) as the inner membrane, and the mycobacterial outer membrane (MOM) made up of longer chain, highly hydrophobic mycolic acids along with other complex lipids, many of which unique to mycobacteria. Between the two is a cell-wall structure of arabinan, galactomannan and peptidoglycan. Long lipoglycans lipomannan (LM) and lipoarabinomannan (LAM) extend from a phosphatidylinositol membrane anchor in the PM out into the periplasm. Finally, a lipid-poor, polysaccharide-rich capsule exists beyond the outer lipid membrane. $Ac_{1/2}PIM_{2/6}$: mono/di- acylated phosphatidylinositol di/hexa-mannoside. AGP: arabinogalactan-peptidoglycan. b: The phospholipid recycling pathway in Mtb. The four classes of phospholipid present in the PM undergo hydrolysis of their fatty acyls catalysed by phospholipases to produce their corresponding polar head groups, or lipid-heads. These are hydrolysed by glycerophosphodiesterase activity into the common product glycerol-3 phosphate, and an alcohol. Glycerol-3 phosphate can be further metabolised by glycerol-3 phosphate phosphatase activity, such as by Rv1692, to glycerol and phosphate, or can be re-acylated to phosphatidic acid (PA) and then activated to cytidine diphosphate-diacylglycerol (CDP-DAG) committing to phospholipid synthesis via subsequent class specific reactions. This pathway enables remodelling of the PM by adjustment in the relative amounts of each phospholipid present. PE: phosphatidylethanolamine, PG: phosphatidylglycerol, PI: phosphatidylinositol, CL: cardiolipin. CdsA: phosphatidate cytidylyltransferase. c: Comparison of a typical PM of a Gram-negative bacteria to that of Mtb. Left- bilayer formed almost exclusively of PE and PG. Right, mycobacterial PM: constructed of four conventional phospholipids PE, PG, PI and CL, with $AcPIM_2$ and $AcPIM_6$ further major components. AcPIMs are unique to actinomycetes. The membrane may be asymmetric, with the inner layer composed predominantly of $AcPIM_2$. These bulky lipids reduce the permeability of the membrane. Trehalose monomycolate (TMM) may also be present in the membrane, and is thought to act as a shuttle for mycolic acids across the PM out into the MOM.

Supplementary Figure 2. Further polar metabolites altered by *glpQ*1 deletion. a: Volcano plot showing the metabolome of Mtb plotted as the fold change in the mean abundance of each feature in Δ*glpQ*1 strain/WT strain, showing positive ion-mode. Means were calculated across replicate cultures: Δ*glpQ*1 = 6 replicates, WT = 8 replicates. This is the corresponding positive mode dataset do the negative mode data plotted in Figure 1a. 1064 features are plotted, 67 of which of significantly altered in abundance. Features in orange are statistically significantly enriched in the Δ*glpQ*1 mutant, features in blue are statistically significantly depleted in the Δ*glpQ*1 mutant. The canonical lipid-heads of Mtb are highlighted in red. The feature in green is glycerophosphocholine $(M+H)^+$ *see Figure 2*. The feature in yellow is GroPThr. b: Plots of the abundance of each of the four canonical lipid-heads of Mtb in extracts from each strain as labelled, plotted as fold changes with the abundance in the WT set to 1. Data is from the amide column chromatography method. For bis(GroP)Gro the abundance in the WT was below the level of detection, so an arbitrary value of 1000 was assigned to allow fold change to be calculated. Bar height represents mean fold change calculated across replicate cultures per strain, error bars the standard deviation, and individual points the value for each replicate within the strain (i.e. the fold change for the individual replicate versus the mean of the WT). c: The structure and MS^2 spectrum for glycerophosphothreonine, seen to accumulate in the Δ*glpQ*1 strain in negative ion-mode and not before described in mycobacteria. Key product ions are annotated with their corresponding proposed fragment structures and their expected masses. 1b was made using some components from Biorender, and can be accessed as BioRender. Gray, R. (2025) https://BioRender.com/gbvyhke and are licensed under CC BY 4.0.

Supplementary Figure 3. Lipidome remodelling in response to *glpQ*1 deletion. a and b show volcano plots showing the lipidome of Mtb plotted as the mean abundance of each ion feature in the Δ*glpQ*1 strain/ in the parent strain. a: positive ion-mode b: negative ion-mode. Total number of features detected is shown, as well as the percentage of features significantly downregulated (blue) and upregulated (orange) in the Δ*glpQ*1 strain. Fold changes are calculated from means across replicate cultures. Representative of three independent experiments. c: Structure of an example mono-acylated

PIM_2 species: $Ac_1PIM_{2(C72)}$. Di-acylated PIMs have a fourth acylation at the hydroxyl group highlighted on the inositol residue. The two acylations of the glycerol do not contribute to the mono- and di-acylation nomenclature, as these form part of the PI anchor and are present in all PIMs.

d: Heat map showing mean fold change in abundance of each individual species of phospholipid in the Δ*glpQ*1 strain versus the parent and in the *ΔglpQ*1::*glpQ*1_*Pimyc* strain versus the parent, log_{10} scale. Red indicates enrichment versus the parent strain, blue depletion. Phospholipid species are arranged into their classes on the x-axis. Individual lipid species are labelled with their class and alkyl form X:Y where X is the combined number of carbons in their alkyl chains and Y is the number of unsaturated bonds. For dimannosylated PIMs, $AcPIM_2$, in the label $Ac_XPIM_{2(CY)}$ X denotes the number of acylations of the inositol / mannose sugars, which can be 1 or 2. In the carbon number in subscript Y refers to the total number of carbons in the species.

Supplementary Figure 4. Repeat glycerophosphocholine sole phosphate source experiment.

Growth profiles of the strains as labelled in Pi-replete (25 mM) and Pi-free (0 mM) media. At day 11 bacteria were transferred from Pi-free media into fresh Pi-replete media or into GroPCho media (0 mM Pi, 25 mM glycerophosphocholine). Duplicate cultures were performed per strain. This is the repeat experiment of the one shown in Figure 2d.

Supplementary Figure 5. Mouse infection studies. a-c. Results of low-dose infection of C57BL/6 mice. Plots of colony forming units per mouse lung (log_{10} scale) for mice infected with either WT (black), the Δ*glpQ*1 strain (red), Δ*glpQ*1::*glpQ*1_*Pimyc* (orange- experiment 2 only) or Δ*glpQ*1::*glpQ*1_*Psmyc* (turquoise- experiment 3 only). 5 mice were sacrificed per timepoint per strain. Points represent means, error bars the standard error of the means. Asterisks shown denote statistical significance of the difference between the CFU/lung for the WT versus Δ*glpQ*1 at that timepoint. (t-test, two tailed, * $p<0.05$, ** $p<0.01$, *** $p<0.001$, ****$p<0.0001$, ns = not significant). There were no

timepoints in any experiment where the CFU/lung was statistically significantly higher for either complement strain versus the Δ*glpQ*1 strain.

d: Thin layer chromatography of PDIM extracts from various strains of H37Rv generated by our laboratory. WT is the parent strain used in all the experiments in this study. 1. is the Δ*glpQ*1 strain, and 5. is the Δ*glpQ*1::*glpQ*1_*Pimyc* strain. 2, 3, 4, and 6 are strains of H37Rv for which no data appears in this study. The rightmost lane (+) contains purified PDIM standard obtained from b.e.i. resources. Note that strains 1 - 3 and 5 - 6 are all progeny of the parent WT strain used in this study. Strain 4 is a further strain generated from a H37Rv stock several years previously.

Supplementary Figure 6. Further features of the phosphate starvation lipidome. a: Total Ion Chromatograms for lipid extracts from WT Mtb cultured in Pi-free media (top) and Pi-replete media (25 mM) (bottom), positive ion-mode. Chromatograms are from single cultures but are highly representative of all replicates across two independent experiments. b: volcano plot showing the lipidome of Mtb plotted as the mean abundance of each ion feature in the WT grown in Pi-free media/ mean abundance in the WT grown in Pi-replete (25 mM) media, negative ion-mode. This is the corresponding negative ion-mode data to the positive ion-mode data plotted in Figure 3a. Representative of 2 independent experiments. PIMs are highlighted in red-purple (horizontal split) and MPMs in red-purple (vertical split). The percentage of features down- and up-regulated are labelled. c: Table showing the mean fold change in abundance of all 70 phospholipids detected, summed by their phospholipid class. For the conventional phospholipids, the area under the curves were summed from positive ion-mode, for PIMs and MPMs negative ion-mode was used as these lipids were not quantifiable in positive ion-mode. Data is from the first of two independent experiments. d. Scatter plots showing the total summed abundance of the 5 species of mono- and di-acylated PIM_2 detected in the lipid extracts of the WT grown in Pi-replete media and in Pi-free media, as indicated. Individual points represent individual replicate cultures, horizontal lines the means across replicates. P values are from 2 tail t-test. Left plot: experiment 1. Right Plot: repeat experiment. e. Chemical structure of an example MPM (β-D-mannosyl phosphomycoketide). Species vary by the length of the saturated oligoisoprenoid chain, which contains

5 methyl branches. f: Scatter plot showing the total summed abundance of the 4 species of MPM detected in the lipid extracts of the WT grown in Pi-replete media and in Pi-free media, as indicated. Individual points represent individual replicate cultures, horizontal lines the means across replicates. P values are from 2 tail t-test. g: EIC overlays for an example of each class of phosphorus-free replacement lipid, with the EICs for each replicate culture of the WT grown in Pi-free media (top) and Pi-replete (25mM) media (bottom) overlain. Scale bars show the intensity of detection in positive ion-mode in counts (arbitrary units). For each plot, the Pi-replete chromatogram is drawn on the same scale as the Pi-free chromatogram.

Supplementary Figure 7. Scheme for the chemical synthesis of GlcA-DAG (16:0/16:0). Reagents and reaction conditions are labelled. See *methods* for full details.

Supplementary Notes One.

Example Collision Induced Dissociation MS2 Spectra

Polar:

GroPEth
$C_5H_{14}NO_6P$
$(M\text{-}H)^-$ m/z 214.0486 (calculated)
14.37_214.0495 (observed)

$(M\text{+}H)^+$ 216.0632 (calculated)
14.39_216.0634 (observed)

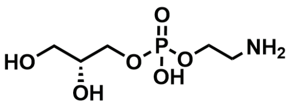

MS2 Negative ESI

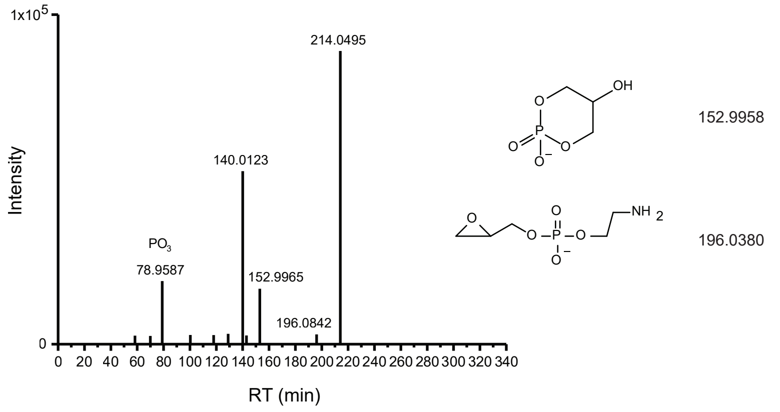

GroPGro
$C_6H_{15}O_8P$
$(M\text{-}H)^-$ 245.0432 (calculated)
13.21_245.0438 (observed)

$(M\text{+}H)^+$ 247.0577 (calculated)
13.21_247.0579 (observed)

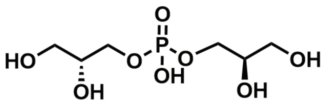

MS2 Negative ESI

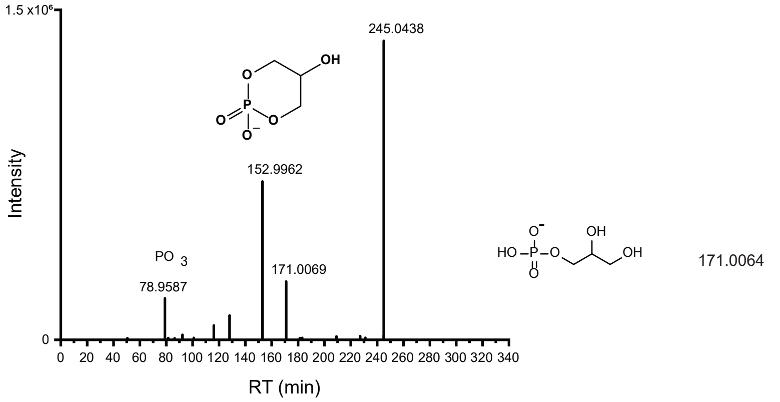

GroPIno
$C_9H_{19}O_{11}P$
$(M\text{-}H)^-$ 333.0592 (calculated)
14.77_333.0593 (observed)

$(M\text{+}H)^+$ 335.0738 (calculated)
14.78_335.0737 (observed)

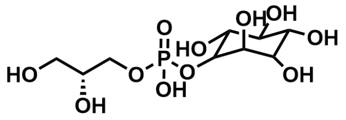

MS2 Negative ESI

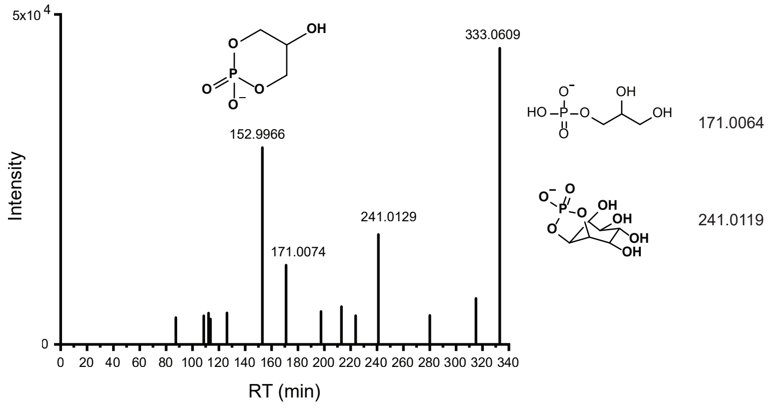

Bis(GroP)Gro
$C_9H_{22}O_{13}P_2$
$(M\text{-}H)^-$ not detected

$(M\text{+}H)^+$ 401.0608 (calculated)
7.36_401.0611 (observed)

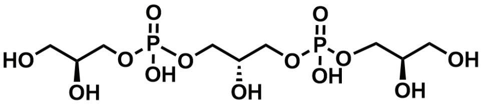

PE (16:0/19:0)
C40H80NO8P
$(M+H)^{+}$ 734.5694 (calculated)
22.13_734.5682 (observed)

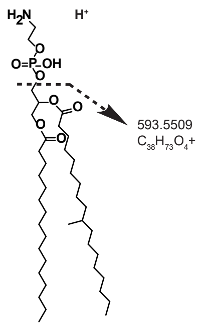

MS2 Positive ESI

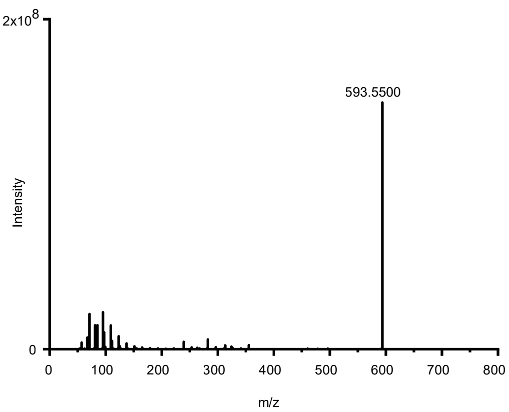

Class identity confirmed by neutral loss of 141 u
from ammoniated or protonated precursor ion,
as per previous publications i.e. Pulfer and Murphy, 2003.

PG (33:0)
$C_{39}H_{77}O_{10}P$
$(M+NH4)^{+}$ 754.5593 (calculated)
18.22_754.5573 (observed)

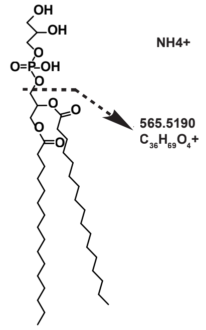

MS2 Positive ESI

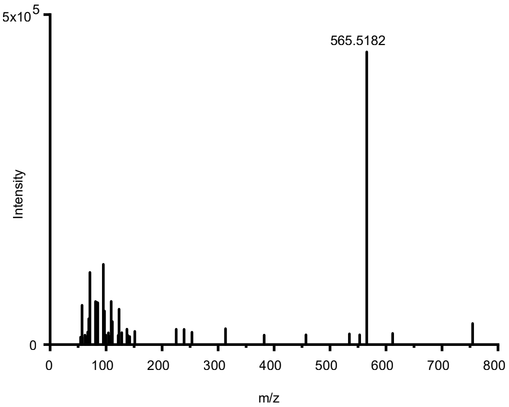

Class identity confirmed by neutral loss of 189 u
(phosphorylglycerol + ammonium) from the ammoniated
precursor ion as per previous publications i.e.Taguchi et al., 2005.

PI (16:0/18:0)
$C_{43}H_{83}O_{13}P$
$(M+NH4)^{+}$ 856.5910 (calculated)
23.01_856.5885 (observed)

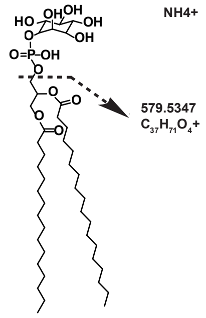

MS2 Positive ESI

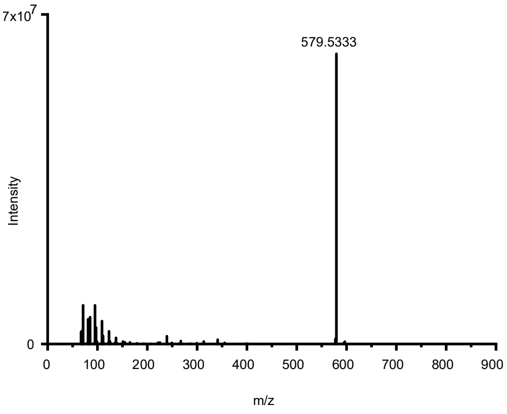

Class identity confirmed by neutral loss of 277 u
(phosphorylinositol + ammonium) from the ammoniated
precursor ion as per i.e.Taguchi et al., 2005.
.

CL (34:1)(36:2)
$C_{79}H_{148}O_{17}P_2$
$(M+NH_4)^{+}$ 1449.0530 (calculated)
Observed as 19.97_1449.0483 (observed)

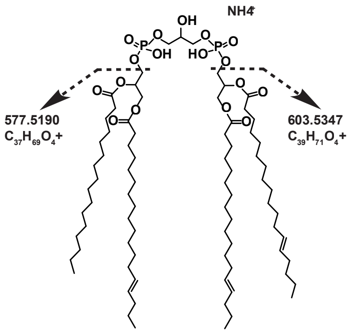

MS2 Positive ESI

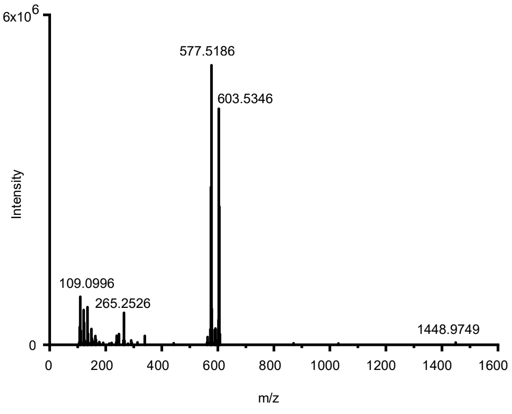

Class identity confirmed by major fragmentation pattern as drawn,
consistent with previous publications i.e. Hsu and Turk, 2006.

Ac₁PIM₂
$C_{72}H_{135}O_{24}P$
$(M-H)^-$ 1413.9008 (calculated)
23.81_1413.8799 (observed)

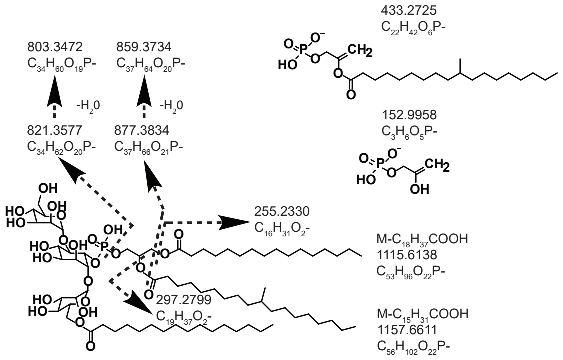

MS2 Negative ESI

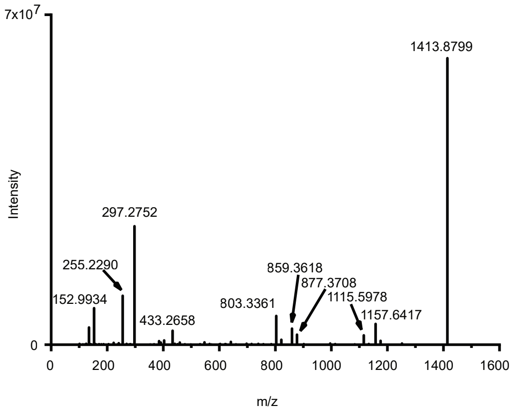

C38 MPM
$C_{38}H_{77}O_9P$
$(M-H)^-$ 707.5232 (calculated)
23.35_707.5206 (observed)

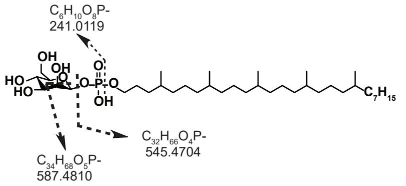

MS2 Negative ESI

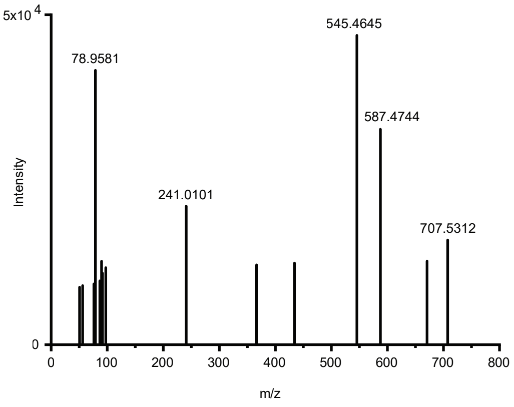

OL 3-OH (22:0/18:1)
$(M+H)^+$, $C_{45}H_{87}N_2O_5$+
735.6610 (calculated)
25.55_735.6586 (observed)

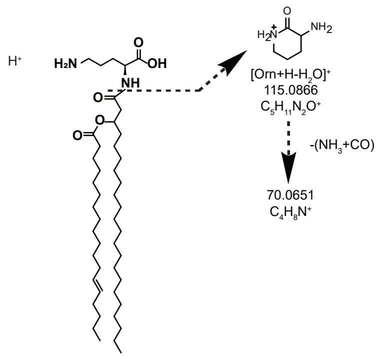

MS2 Positive ESI

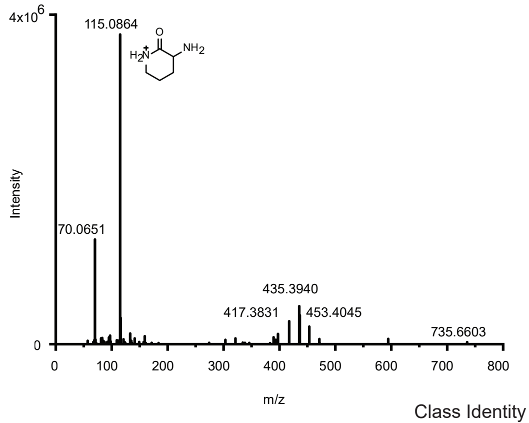

Class Identity confirmed by 115 and 70 u derivatives of the ornithine headgroup, as per previous publications i.e. Zhang et al 2009

GlcA DAG (19:0/16:0)
$(M+NH_4)^+$, $C_{44}H_{86}NO_{11}$+
804.6195 (calculated)
21.78_804.6174 (observed)

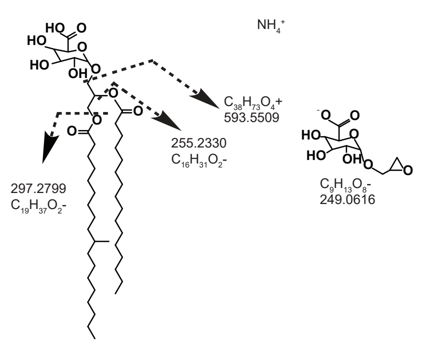

MS2 Positive ESI

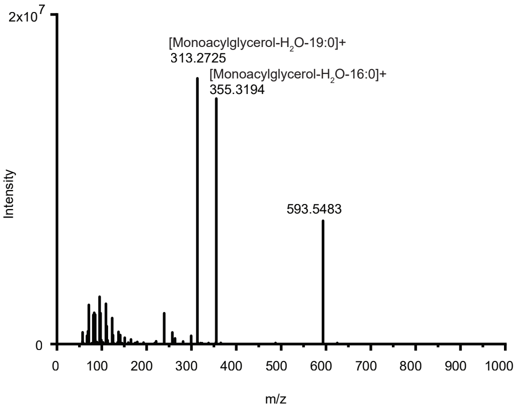

MS2 Negative ESI

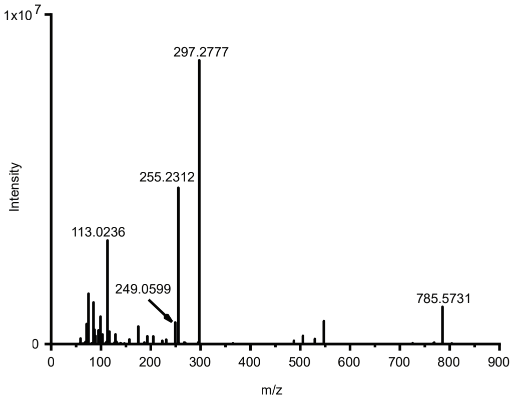

Class Identity confirmed by neutral loss of 211 u (glucuronic acid + ammonium) and by the 249 u oxirane-containing product ion, as per previous publications i.e. Semeniuk et al 2014.

Me-GlcA DAG (19:0/16:0)
$(M+NH_4)^+$, $C_{45}H_{88}NO_{11}$+
818.6352 (calculated)
4.42_818.6326 (observed)

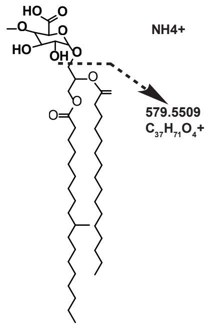

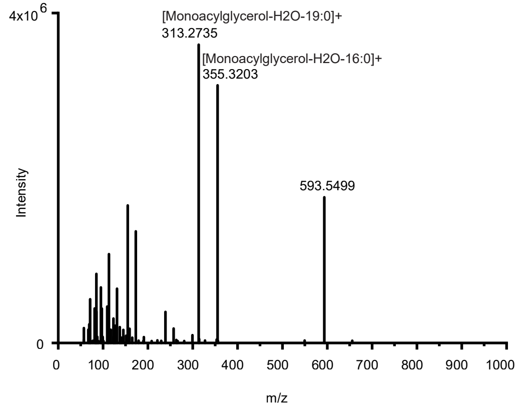

Class identity surmised by neutral loss of 225 u (O-methyl glucuronic acid + ammonium).

TMM C78 alpha
$(M+NH_4)$+, $C_{90}H_{176}NO_{13}$+
1479.3136 (calculated)
8.68_1479.3135 (observed)

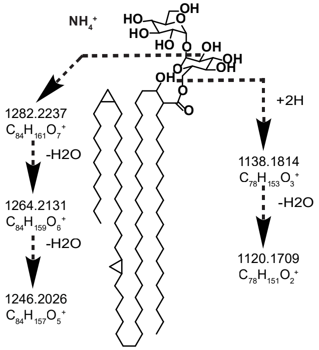

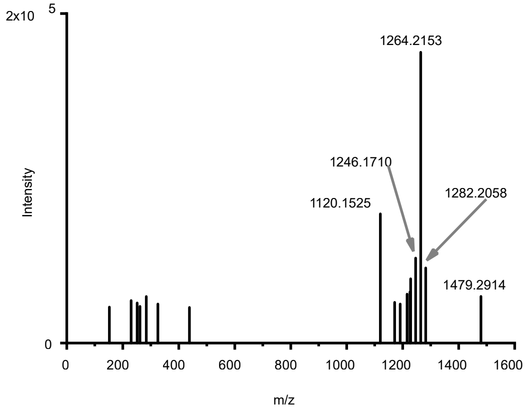

Class identity confirmed via the fragmentation pattern shown, consistent with previous publications i.e. Layre et al 2011.

Supplementary Notes 2

MS signals for phospholipid alkylform families. For normal phosphate experiments. (relating to figure 1c). Signals shown are from a representative pooled biological control sample. Labels are of the detected m/z value. See Supplementary Data 2 for further details.

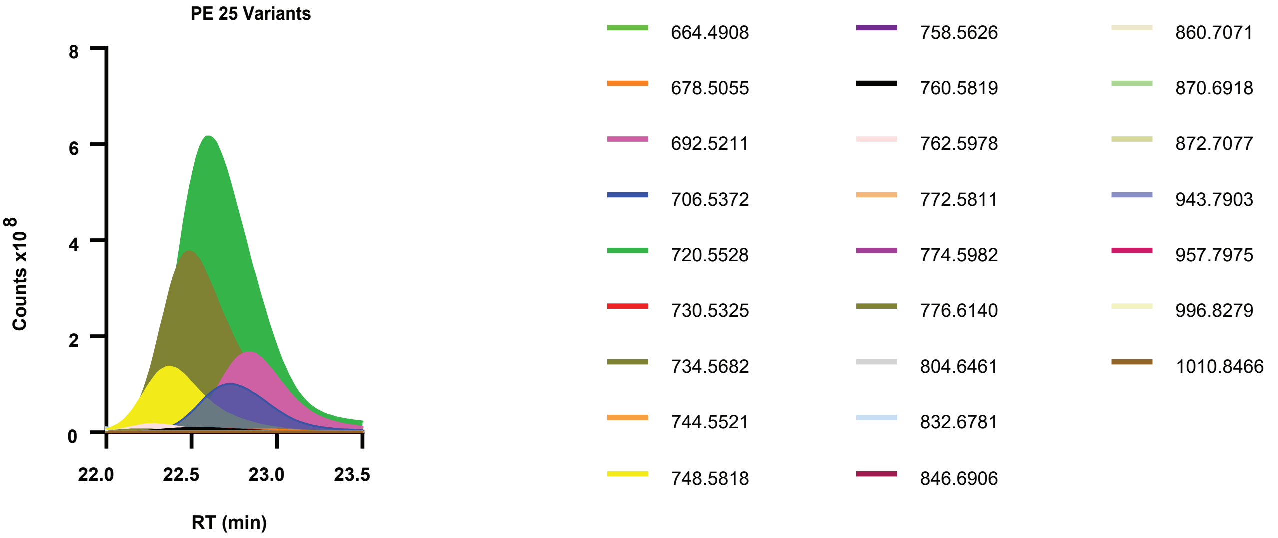

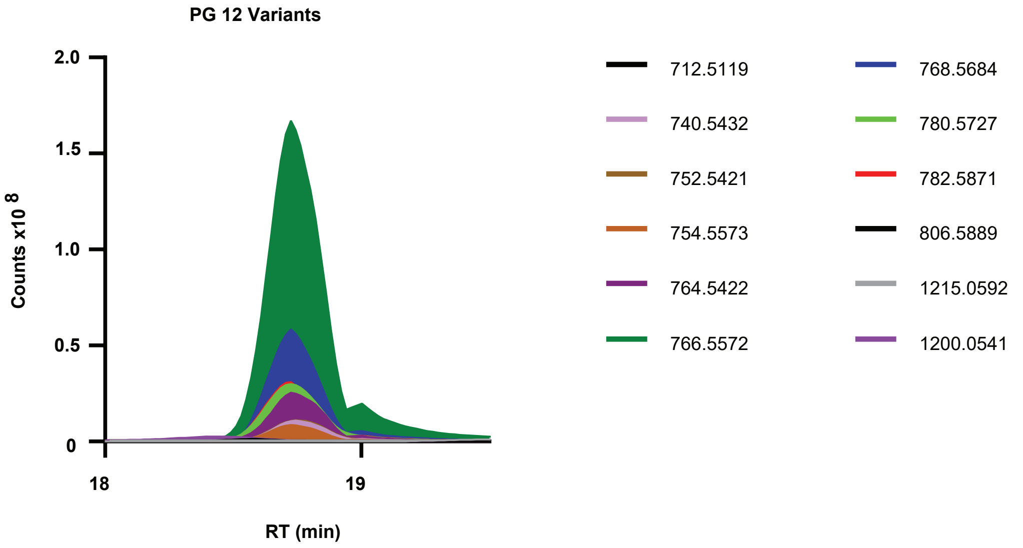

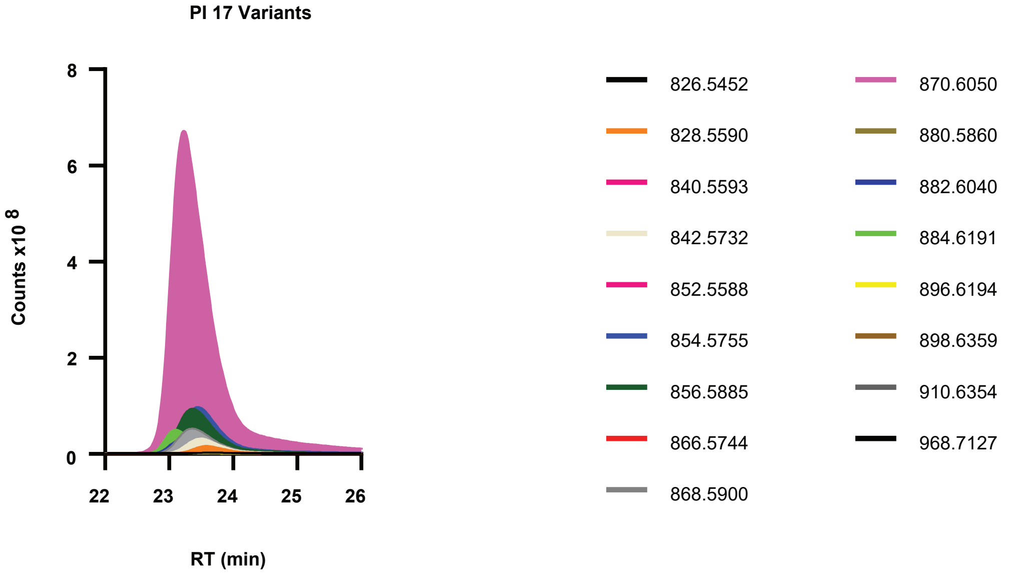

Supplementary Notes 2 (continued)

MS signals for phospholipid alkylform families for normal phosphate experiments (relating to figure 1c).

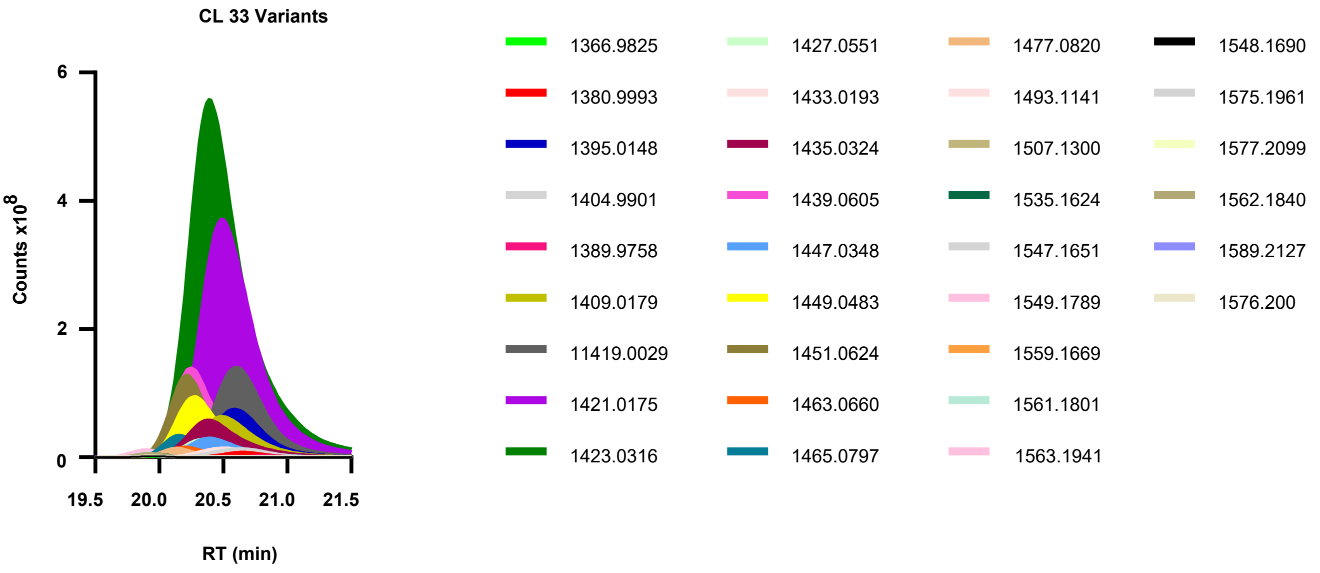

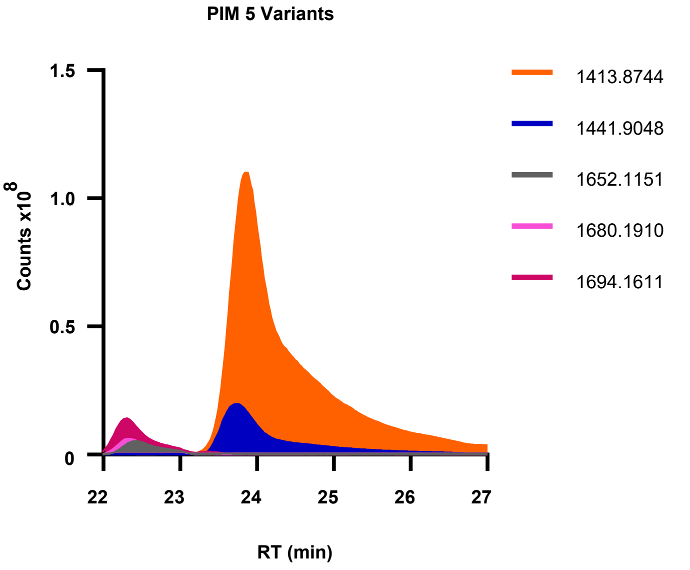

SUPPLEMENTARY MATERIALS REFERENCES

PULFER, M. & MURPHY, R. C. 2003. Electrospray mass spectrometry of phospholipids. Mass Spectrom Rev, 22, 332-64.

TAGUCHI, R., HOUJOU, T., NAKANISHI, H., YAMAZAKI, T., ISHIDA, M., IMAGAWA, M. & SHIMIZU, T. 2005. Focused lipidomics by tandem mass spectrometry.
J Chromatogr B Analyt Technol Biomed Life Sci, 823, 26-36.

HSU, F. F. & TURK, J. 2006. Characterization of cardiolipin as the sodiated ions by positive-ion electrospray ionization with multiple stage quadrupole ion-trap mass spectrometry.
J Am Soc Mass Spectrom, 17, 1146-57.

ZHANG, X., FERGUSON-MILLER, S. M. & REID, G. E. 2009. Characterization of ornithine and glutamine lipids extracted from cell membranes of Rhodobacter sphaeroides.
J Am Soc Mass Spectrom, 20, 198-212.

SEMENIUK, A., SOHLENKAMP, C., DUDA, K. & HOLZL, G. 2014. A bifunctional glycosyltransferase from Agrobacterium tumefaciens synthesizes monoglucosyl and glucuronosyldiacylglycerol under phosphate deprivation. J Biol Chem, 289, 10104-14.

LAYRE, E., SWEET, L., HONG, S., MADIGAN, C. A., DESJARDINS, D., YOUNG, D. C., CHENG, T. Y., ANNAND, J. W., KIM, K., SHAMPUTA, I. C., MCCONNELL, M. J.,DEBONO, C. A., BEHAR, S. M., MINNAARD, A. J., MURRAY, M., BARRY, C. E., 3RD, MATSUNAGA, I. & MOODY, D. B. 2011.
A comparative lipidomics platform for chemotaxonomic analysis of Mycobacterium tuberculosis. Chem Biol, 18, 1537-49.
