# Supplementary material for: Mycobacterium tuberculosis overcomes phosphate starvation by extensively remodelling its lipidome with phosphorus-free lipids
Source: Nat Commun. 2025 Nov 20;16:11317. doi: 10.1038/s41467-025-66437-w (PMC12722247; doi:10.1038/s41467-025-66437-w)
Supplement: Supplementary file 2 — Description of Additional Supplementary File [file 41467_2025_66437_MOESM2_ESM.pdf]

Description of Additional Supplementary Files

Supplementary Data 1: Ion List_Polar Heads Table of the ions identified as phospholipid heads in the polar LC-MS experiments. NB. Bisglycerophosphoglycerol (Bis(GroP)Gro) data is from the amide LC-MS method, whereas all others are from the HILIC method.

Supplementary Data 2: Ion List_Lipids in unmodified 7H9 Table of the ions identified as phospholipids in the apolar LC-MS experiments in normal phosphate culture (25mM).

Supplementary Data 3: Ion List_zero vs 25mM phosphate lipids Table of the ions of phospholipids and phosphorus-free lipids as identified in the apolar LC-MS experiments of zero phosphate culture versus replete (25mM) phosphate culture.
