# Supplementary material for: Mycobacterium tuberculosis overcomes phosphate starvation by extensively remodelling its lipidome with phosphorus-free lipids
Source: Nat Commun. 2025 Nov 20;16:11317. doi: 10.1038/s41467-025-66437-w (PMC12722247; doi:10.1038/s41467-025-66437-w)
Supplement: Supplementary file 6 — Reporting summary [file 41467_2025_66437_MOESM6_ESM.pdf]

nature portfolio

Corresponding author(s): Robert Gray and Luiz Pedro S. de Carvalho

Last updated by author(s): 06/10/2025

# Reporting Summary

Nature Portfolio wishes to improve the reproducibility of the work that we publish. This form provides structure for consistency and transparency in reporting. For further information on Nature Portfolio policies, see our Editorial Policies and the Editorial Policy Checklist.

## Statistics

For all statistical analyses, confirm that the following items are present in the figure legend, table legend, main text, or Methods section.

| n/a | Confirmed | |
|---|---|---|
| ☐ | ☒ | The exact sample size (*n*) for each experimental group/condition, given as a discrete number and unit of measurement |
| ☐ | ☒ | A statement on whether measurements were taken from distinct samples or whether the same sample was measured repeatedly |
| ☐ | ☒ | The statistical test(s) used AND whether they are one- or two-sided<br>*Only common tests should be described solely by name; describe more complex techniques in the Methods section.* |
| ☒ | ☐ | A description of all covariates tested |
| ☐ | ☒ | A description of any assumptions or corrections, such as tests of normality and adjustment for multiple comparisons |
| ☐ | ☒ | A full description of the statistical parameters including central tendency (e.g. means) or other basic estimates (e.g. regression coefficient) AND variation (e.g. standard deviation) or associated estimates of uncertainty (e.g. confidence intervals) |
| ☐ | ☒ | For null hypothesis testing, the test statistic (e.g. *F*, *t*, *r*) with confidence intervals, effect sizes, degrees of freedom and *P* value noted<br>*Give P values as exact values whenever suitable.* |
| ☒ | ☐ | For Bayesian analysis, information on the choice of priors and Markov chain Monte Carlo settings |
| ☒ | ☐ | For hierarchical and complex designs, identification of the appropriate level for tests and full reporting of outcomes |
| ☒ | ☐ | Estimates of effect sizes (e.g. Cohen's *d*, Pearson's *r*), indicating how they were calculated |

*Our web collection on statistics for biologists contains articles on many of the points above.*

## Software and code

Policy information about availability of computer code

Data collection

no custom code was used
LC-MS data was acquired using Xcalibur 3.0.63 software (Thermo Scientific)
Electron Microscopy:
Cryo-EM images were collected on a Talos Arctica microscope (Thermo Fisher Scientific, USA) using Tomography software (v5.21, Thermo Fisher Scientific, USA) at magnification corresponding to 3.25 Å pixel size and a nominal defocus range−8-−4µm. Images were recorded using a Falcon III camera (Thermo Fisher Scientific, USA).
The movies were imported into Relion (v 5.0.0) 76, followed by beam-induced motion correction using Relion's implementation of the UCSF motioncor2 program for whole-frame movie alignment, and CTF estimation using CTFFIND v4.1 77.

Data analysis

no code was used to analyse the data.
The following software was used in data analysis:
Microsoft Excel
Progenesis QI v3 (Nonlinear Dynamics)
Thermo Scientific Freestyle 1.8 SP2
Origin 2025
GraphPad Prism version 10.1.1
Electron Microscopy: image analysis and quantification:
Micrographs were opened using Fiji/Image J (Version:2.16.0/1.54p)

For manuscripts utilizing custom algorithms or software that are central to the research but not yet described in published literature, software must be made available to editors and reviewers. We strongly encourage code deposition in a community repository (e.g. GitHub). See the Nature Portfolio guidelines for submitting code & software for further information.

## Data

Policy information about availability of data

All manuscripts must include a data availability statement. This statement should provide the following information, where applicable:

- Accession codes, unique identifiers, or web links for publicly available datasets
- A description of any restrictions on data availability
- For clinical datasets or third party data, please ensure that the statement adheres to our policy

The LC-MS datasets generated and analysed during the current study are available in the Francis Crick Institute Figshare platform, at DOI: 10.25418/crick.29646266. Additional source data for figures 1-5 are provided with the paper.

## Research involving human participants, their data, or biological material

Policy information about studies with human participants or human data. See also policy information about sex, gender (identity/presentation), and sexual orientation and race, ethnicity and racism.

| | |
|---|---|
| Reporting on sex and gender | There were nil human participants in this study |
| Reporting on race, ethnicity, or other socially relevant groupings | N/A |
| Population characteristics | N/A |
| Recruitment | N/A |
| Ethics oversight | N/A |

Note that full information on the approval of the study protocol must also be provided in the manuscript.

# Field-specific reporting

Please select the one below that is the best fit for your research. If you are not sure, read the appropriate sections before making your selection.

☒ Life sciences ☐ Behavioural & social sciences ☐ Ecological, evolutionary & environmental sciences

For a reference copy of the document with all sections, see nature.com/documents/nr-reporting-summary-flat.pdf

# Life sciences study design

All studies must disclose on these points even when the disclosure is negative.

| | |
|---|---|
| Sample size | No sample size calculations were performed. LC-MS studies contained 5-6 replicate cultures as this is in our experience an effective balance between generating data which will show true biological differences with appropriate statistical significance, and minimising unnecessary processing of specimen which pose a biosafety hazard, such as M. tuberculosis samples. Mouse infection studies were limited to 5 mice per data point per condition for ethical purposes. |
| Data exclusions | No data was excluded, except for the following: LC-MS samples were excluded if their normalisation factor (normalisation to total ion intensity) was greater than 2 fold. For volcano plots, as stated above, features failing the CV < 30 statistical filter were not included in the plots, but may have been further interrogated. For the extracted ion chromatogram overlay in Figure 2b, as described in the figure legend, 2 outliers were not included in the overlay plot, but their values are included in the calculations of statistical significance for the differences between the strains, to prevent bias. |
| Replication | Typically, experiments are performed at least in triplicate (three biological replicates) and repeated twice independently. All attempts at replication were essentially successful: where data or fidnings varied between replicates these are stated in the manuscript: i.e. see the discussion on the variation in the degree of attenuation in the mouse studies, |
| Randomization | LC/MS samples are randomized prior analysis. |
| Blinding | Blinding was not performed. All measurements were automated or quantitative, so were not susceptible to investigator bias at the point of measurement. |

# Reporting for specific materials, systems and methods

We require information from authors about some types of materials, experimental systems and methods used in many studies. Here, indicate whether each material, system or method listed is relevant to your study. If you are not sure if a list item applies to your research, read the appropriate section before selecting a response.

## Materials & experimental systems

| n/a | Involved in the study |
|---|---|
| ☒ | ☐ Antibodies |
| ☒ | ☐ Eukaryotic cell lines |
| ☒ | ☐ Palaeontology and archaeology |
| ☐ | ☒ Animals and other organisms |
| ☒ | ☐ Clinical data |
| ☒ | ☐ Dual use research of concern |
| ☒ | ☐ Plants |

## Methods

| n/a | Involved in the study |
|---|---|
| ☒ | ☐ ChIP-seq |
| ☒ | ☐ Flow cytometry |
| ☒ | ☐ MRI-based neuroimaging |

## Animals and other research organisms

Policy information about studies involving animals; ARRIVE guidelines recommended for reporting animal research, and Sex and Gender in Research

| | |
|---|---|
| Laboratory animals | Six to eight week old C57BL/6J female mice were used for all experiments, these were bred and housed under specific pathogen-free conditions in the Biological Research Facility at the Francis Crick Institute. |
| Wild animals | The study did not involve wild animals |
| Reporting on sex | Use of female mice are the standard in the field. |
| Field-collected samples | The study did not involve samples collected from the field. |
| Ethics oversight | All infection studies were approved by the Francis Crick Institute Ethics Committee and performed under a UK Home Office approved Animal License (P4D8F6075). C57BL/6 mice were bred and maintained in the Biological Research Facility at the Francis Crick Institute. Procedures involving mice were performed in strict accordance with the United Kingdom Animals (Scientific Procedures) Act 1986 and the Institute's policies on the Care, Welfare and Treatment of Animals. |

Note that full information on the approval of the study protocol must also be provided in the manuscript.

## Plants

| | |
|---|---|
| Seed stocks | N/A |
| Novel plant genotypes | N/A |
| Authentication | N/A |
